# Cancer Cells Expressing Oncogenic Rat Sarcoma Show Drug-Addiction Toward Epidermal Growth Factor Receptor Antibodies Mediated by Sustained MAPK Signaling

**DOI:** 10.3389/fonc.2019.01559

**Published:** 2020-01-21

**Authors:** Joseph Tintelnot, Sina Metz, Marie Trentmann, Anna Oberle, Lisa von Wenserski, Christoph Schultheiß, Friederike Braig, Malte Kriegs, Boris Fehse, Kristoffer Riecken, Carsten Bokemeyer, Alexander Stein, Mascha Binder

**Affiliations:** ^1^Department of Oncology and Hematology, BMT With Section Pneumology, University Medical Center Hamburg-Eppendorf, Hamburg, Germany; ^2^Department of Internal Medicine IV, Oncology/Hematology, Martin-Luther-University Halle-Wittenberg, Halle (Saale), Germany; ^3^Radiation Biology and Radiooncology, University Medical Center Hamburg-Eppendorf, Hamburg, Germany; ^4^UCCH Kinomics Core Facility, University Medical Center Hamburg-Eppendorf, Hamburg, Germany; ^5^Research Department Cell and Gene Therapy, Department of Stem Cell Transplantation, University Medical Center Hamburg-Eppendorf, Hamburg, Germany

**Keywords:** EGFR antibodies, colorectal cancer, oncogenic RAS, MAPK signaling, acquired resistance

## Abstract

Epidermal growth factor receptor (EGFR) antibodies may have detrimental effects in patients with metastatic colorectal cancer expressing oncogenic Rat sarcoma (RAS). Since a significant number of patients acquire RAS-mediated resistance during EGFR-directed treatment, understanding the molecular mechanism underlying these antibody-mediated tumor-promoting effects is of relevance to design more resistance-preventive treatment approaches. To test this, we set up a Ba/F3 cellular model system transformed to EGFR/RAS dependency to be able to study proliferation, RAS activity as well as MAPK signaling upon inhibition of wild-type RAS isoforms by therapeutic EGFR antibodies. Here, we show that the EGFR antibodies cetuximab and panitumumab induce paradoxical stimulation and enhance proliferation in cells expressing oncogenic RAS (KRAS G12V). These experiments clearly showed that the stimulatory effect is a direct result of the antibody-EGFR interaction leading to prolonged mitogen-activated protein-Kinase (MAPK) signaling. The effect was also induced by antibody-chemotherapy combinations but always depended on simultaneous low-level ligand-dependent EGFR pathway activation. Moreover, we observed significant growth retardation of RAS mutant cells after antibody withdrawal compatible with a drug-addiction phenotype. Our data suggests that EGFR antibodies paradoxically sustain MAPK signaling downstream of oncogenic RAS thereby driving proliferation of RAS mutant tumors or tumor subclones. The observed drug-addiction encourages fixed-duration or liquid-biopsy-guided drug holiday concepts to preventively clear RAS mutant subclones selected under EGFR-directed therapeutic pressure.

## Introduction

Monoclonal antibodies which inhibit signaling downstream the epidermal growth factor receptor (EGFR) have become one of the mainstays of targeted therapy in metastatic colorectal cancer (mCRC) (1). Both the chimeric EGFR antibody cetuximab and the fully human antibody panitumumab have been approved as single agents or in combination with chemotherapy (2–9). Resistance to these antibodies can be mediated by mutations in downstream signaling molecules such as RAS (10–13), which remains to date the only validated and widely accepted molecular marker that predicts lack of response to EGFR antibodies (11, 14–16). Since mCRC evolves by a reiterative process of genetic diversification and clonal evolution under the selective pressure of repetitive therapeutic challenge, activating mutations in RAS are not only a primary mechanism of resistance, but can mediate acquired resistance in the course of EGFR antibody treatment (17, 18).

Even of more concern than the–expected–lack of benefit of EGFR antibodies is a suspected harmful effect of this treatment in the subset of patients with primary or acquired RAS mutations. A number of clinical trials have demonstrated inferior outcomes of EGFR-antibody treated patients with RAS mutations compared to treatment with the chemotherapy backbone alone. This effect was found for cetuximab and panitumumab in the context of an oxaliplatinum-containing chemotherapy regimen (14, 19), but also for panitumumab in combination with irinotecan (20). Interestingly, inferior outcomes were not observed in patients with RAS mutant tumors in other phase III trials of panitumumab (6, 21). Moreover, a liquid biopsy trial showed that RAS mutant subclones selected on EGFR-directed therapeutic pressure decrease in size once the selecting antibody was withdrawn, suggesting a fitness disadvantage, or some sort of dependency of those clones on the drug (22).

Yet, the mechanisms underlying the tumor-promoting effects of EGFR antibodies observed in patients with RAS mutant tumors remain largely unclear. A growing body of evidence suggests that loss of (co-existing) wild-type RAS isoforms in tumors harboring an activating oncogenic KRAS, NRAS, or HRAS mutation enhances cellular fitness. This has been shown for KRAS-mutant acute myeloid leukemia, colon and lung cancer cells that lost the remaining (tumor-suppressive) wild-type KRAS allele (23, 24). Silencing experiments of wild-type RAS isoforms show enhanced ERK phosphorylation suggesting that there may be an inhibitory effect of the wild-type isoform on the oncogenic RAS isoform (25). Ambrogio et al. (26) further refine this concept by specifying that the inhibitory effect exerted by wild-type KRAS is dependent on its dimerization with mutant KRAS.

Here, we hypothesized that not only loss of wild-type RAS, but also pharmacological inhibition of wild-type RAS by EGFR-inhibiting antibodies such as cetuximab or panitumumab may remove the inhibitory effect of wild-type RAS on mutant RAS. This, in turn, would lead to enhanced downstream signaling and proliferation and could explain the detrimental effect of EGFR antibodies in the context of an oncogenic RAS mutation. To test this, we set up a Ba/F3 cellular model system transformed to EGFR/RAS dependency to be able to study proliferation, RAS activity as well as MAPK signaling upon inhibition of wild-type RAS isoforms by therapeutic EGFR antibodies. This model was very well-suited for the purpose of this trial since it allowed to measure meaningful effects of the therapeutic antibodies in a context that is strictly dependent on the EGFR pathway. In line with our theory, we found increased MAPK signaling as well as enhanced proliferation of RAS mutant cells upon EGFR inhibition in the presence of EGF. These findings may explain the detrimental effect of EGFR inhibition on patients with RAS mutant mCRC and once more stress the importance of all-RAS mutational testing before treatment initiation. Drug holiday concepts should be evaluated in future clinical trials to prevent the selection of RAS mutant clones and to re-sensitize toward EGFR inhibiting antibodies.

## Materials and Methods

Please refer to detailed methods section in the supplementary part of the manuscript (Supplementary Data Sheet 1).

### Ba/F3 Cellular Model System

Murine IL-3-dependent Ba/F3 cells were lentivirally transduced with different combinations of wild-type or mutant human EGFR (hEGFR) and/or wild-type or mutant human KRAS (hKRAS) encoding vectors as indicated and established as stable cell lines after cell sorting and antibiotic selection followed by flow cytometric characterization and typing of murine RAS status by next-generation sequencing (27). Cells were cultured on IL-3 or in the presence of human EGF (hEGF).

### Cellular Drug-Sensitivity and Proliferation Assays

Cell growth was measured by trypan blue cell counting using Vi-CELL Cell Viability Analyzer (Beckman Coulter, Brea, USA) or WST-8 assay (cell counting kit-8, Sigma-Aldrich, St. Louis, USA) in the presence and absence of ligands and/or therapeutics (antibodies, chemotherapy).

### Signaling Analyses

The differential kinase activity between Ba/F3 hEGFR wt / hKRAS G12V cells treated with hEGF vs. hEGF + cetuximab was estimated using a PamGene serine/threonine Chip according to the manufacturer's instructions. Conventional Western Blots were performed to assess ERK1/2 phosphorylation status. GTP-RAS loading was performed via pull-down assay (PR-950, JenaBioscience, Jena, Germany).

### Statistics

Student's *t*-tests and ANOVA analyzes were calculated using GraphPad Prism version 7.00 (GraphPad Software, La Jolla California USA).

## Results

### Set-Up and Functional Validation of Ba/F3 Cellular Model System Engineered to Express EGFR and Oncogenic RAS

To investigate the functional consequences of EGFR pathway inhibition by monoclonal antibodies in cells harboring activating oncogenic RAS mutations, we explored colon cancer cell lines as a potential model system. The KRAS G12V-positive cell line SW480 and its lymph node derivative SW620, the KRAS wild-type SW48 cell line and the DLD-1 cell line carrying a KRAS G13V mutation were cultured in the absence and presence of human EGF (hEGF). As shown in Supplementary Figure 1, addition of hEGF did not increase proliferation of both the RAS mutant and unmutated cell lines, suggesting that these lines grow virtually independent of the EGFR ligand.

**Figure 1:**
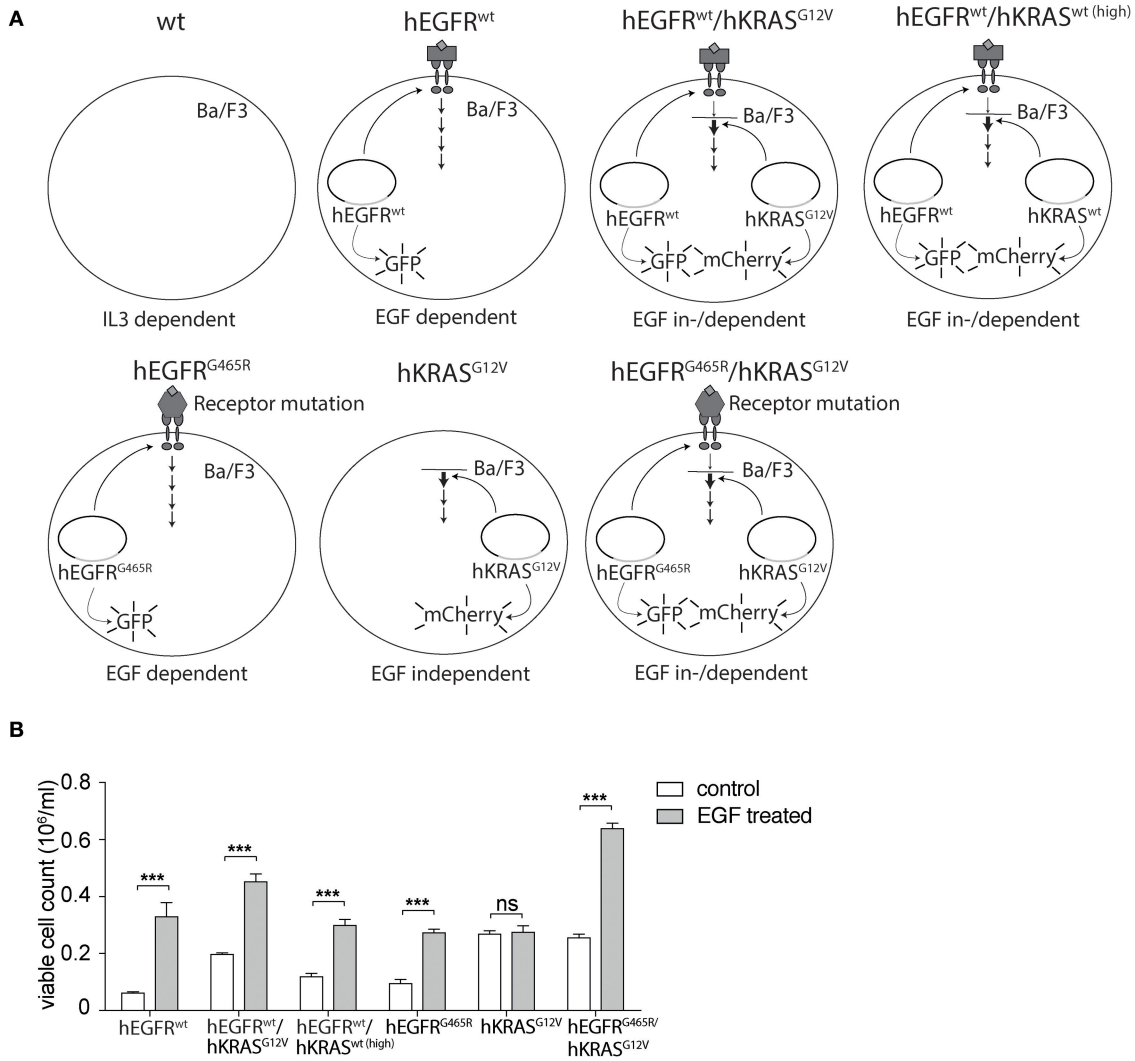
Set-up of hEGFR- and hKRAS-transducible hEGF-dependent Ba/F3 cellular model system. **(A)** Schematic overview of cellular Ba/F3 model system. Different Ba/F3 model cell lines were transduced with LeGO vectors to express hEGFR wild-type or G465R mutant genes (cDNAs) in conjunction with eGFP and/or the hKRAS wild-type or G12V mutant in combination with mCherry. **(B)** Growth properties of different Ba/F3 cell lines in absence and presence of hEGF. Proliferation of hEGFR wt, hEGFR wt / hKRAS wt (high), hEGFR wt / hKRAS G12V, hEGFR G465R, hKRAS G12V, and hEGFR G465R / hKRAS G12V transduced Ba/F3 cells was assessed in the absence and presence of hEGF. Cells were seeded in triplicate at equal densities and the average number of viable cells was measured after 72 h by trypan blue exclusion using Vi-CELL Cell Viability Analyzer. Experiments were performed three times with (*n* = 3). Results of one representative experiment are represented as mean ± SD. Statistical significance was calculated using 2-way ANOVA followed by a Sidak *post-hoc* test for multiple comparison (***p < 0.001).

In search for a more suitable EGF-dependent cellular model system, we engineered the murine EGFR-negative, IL3-dependent pro-B cell line Ba/F3 to stably express the human EGFR (hEGFR) or its mutant variant hEGFR G465R, that harbors an EGFR ectodomain mutation conferring resistance to EGFR-inhibiting antibodies cetuximab and panitumumab, using lentiviral expression vectors as previously described (27, 28). Furthermore, we transduced these cells with a lentiviral vector encoding the human KRAS gene (hKRAS) or its oncogenic codon 12 GGT>GTT mutant (G12V). For further experiments only cells expressing moderate levels of mutant hKRAS as seen in mCherry expression levels and western blotting (Supplementary Figure 2) were chosen to ensure that excessive overexpression of the protein does not confound our results. All engineered variants are shown in Figure 1A. Ectopic expression of hEGFR wt or hEGFR G465R resulted in IL3-independent growth upon stimulation with hEGF as described previously, but did not grow without hEGF (Figure 1B) (28). In contrast, hEGFR wt / hKRAS wt (high) and hEGFR wt / hKRAS G12V transduced Ba/F3 cells showed modest and highly increased proliferation without hEGF addition, suggesting growth factor independence conferred by RAS wild-type overexpression or the oncogenic RAS mutation (Figure 1B). However, stimulation with hEGF further enhanced proliferation of these cells, which was absent in cells transduced with hKRAS G12V only (Figure 1B). Since hEGFR wt / hKRAS wt (high) transduced Ba/F3 cells showed decreased proliferation at later time points in the growth curve, which was due to feedback EGFR receptor downregulation (Supplementary Figure 3), we excluded these cells from further experiments.

### Cetuximab and Panitumumab Paradoxically Enhance Proliferation of Cells Harboring an Oncogenic RAS Mutation in the Presence of hEGF

**Figure 2:**
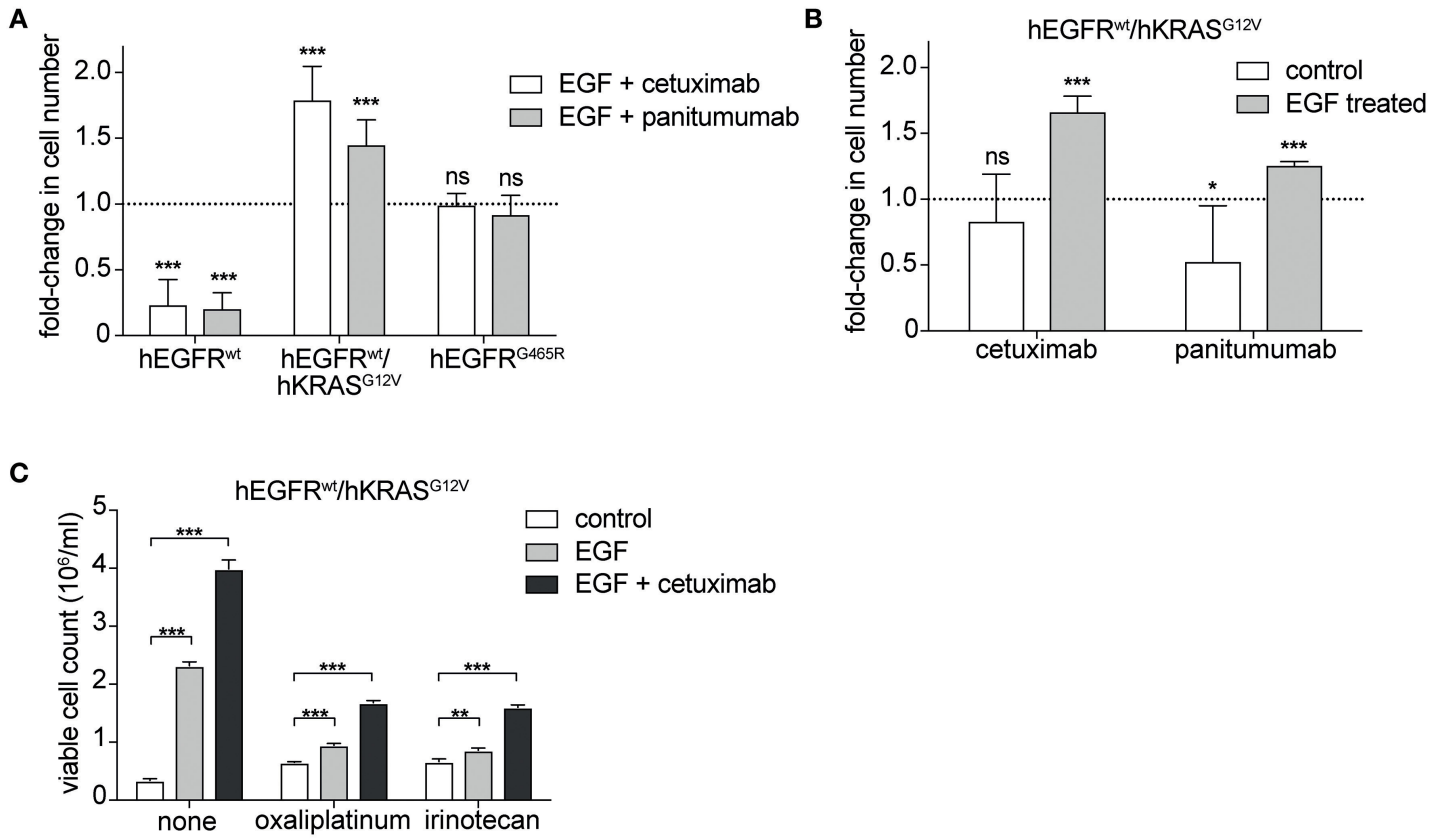
Cetuximab and panitumumab drive proliferation of RAS mutant cells in the presence of EGF. **(A)** hEGFR wt / hKRAS G12V are paradoxically stimulated by EGFR-targeting antibody in the presence of hEGF. hEGFR wt, hEGFR wt / hKRAS G12V, or hEGFR G465R transduced Ba/F3 cells were seeded in triplicates at equal densities and treated with hEGF in combination with an EGFR-targeting antibody as indicated. Proliferation was assessed by counting the average number of viable cells every 24 h for 7 days using Vi-CELL Cell Viability Analyzer after trypan blue staining. For each treatment, data is expressed as the fraction of maximal cell count at 168 h normalized to the hEGF-stimulated control. Data is represented as mean ± SD with (*n* = 6). Statistical significance was calculated by t-test (***p < 0.001; ns, not significant). **(B)** Stimulatory antibody effect is present only upon engagement of the hEGFR signaling pathway by hEGF in hEGFR wt / hKRAS G12V Ba/F3 cells. Proliferation of hEGFR wt / hKRAS G12V transduced Ba/F3 cells was assessed in the absence or presence of hEGF plus cetuximab or panitumumab as indicated. Cells were seeded in triplicates at equal densities and the average number of viable cells was measured by trypan blue staining every 24 h for 7 days using Vi-CELL Cell Viability Analyzer. For each treatment, data is expressed as the fraction of maximal cell count at 168 h normalized to its respective control. Experiments were performed two times and results are represented as mean ± SD with (*n* = 6). Statistical significance was calculated using unpaired student's t-test (***p < 0.001; *p < 0.05; ns: not significant). **(C)** Stimulatory antibody effect persists in the presence of oxaliplatinum and irinotecan. hEGFR wt / hKRAS G12V transduced Ba/F3 cells were treated with hEGF, cetuximab and oxaliplatinum or irinotecan (IC50 dosing) as indicated. Cells were seeded in triplicates at equal densities and the average number of viable cells was measured by trypan blue staining every 24 h for 7 days using Vi-CELL Cell Viability Analyzer. For each treatment, data is expressed as viable cell count at 168 h. The experiment was performed in triplicates and results are represented as means ± SD with (*n* = 3). Statistical significance was calculated using 2-way ANOVA followed by a Tukey *post-hoc* test for multiple comparison (**p < 0.001).

Next, we tested the sensitivity of hEGFR wt, hEGFR G465R, and hEGFR wt / hKRAS G12V transduced Ba/F3 cells to cetuximab and panitumumab. Treatment with any of the EGFR-targeting antibodies effectively decreased proliferation of Ba/F3 cells transduced with hEGFR wt, but not of those expressing hEGFR G465R or hEGFR wt / hKRAS G12V (Figure 2A). Interestingly, antibody treatment of Ba/F3 cells transduced with hEGFR wt / hKRAS G12V not only resulted in a lack of growth inhibition but induced a substantial stimulatory effect. Further proliferation assays showed that such paradoxical antibody stimulation occurred only in the presence of both antibody and growth factor, but not in the absence of growth factor (Figure 2B). Due to its higher affinity, panitumumab was initially used at half the cetuximab concentration (29, 30). Under these conditions, the stimulatory effect was comparable to the one induced by cetuximab. In a larger titration experiment with panitumumab, we observed that the stimulatory effect was dose-dependent with higher concentrations (that completely outcompeted hEGF) showing lesser stimulation as shown in Supplementary Figure 4. Based on this data, we postulated that cetuximab and panitumumab paradoxically drive proliferation of RAS mutant cells only under conditions of simultaneous ligand-dependent EGFR pathway activation. This could explain that such paradoxically stimulation was not seen with the highly potent EGFR inhibitor Erlotinib, even at very low doses (data not shown).

Since detrimental antibody effects have been observed in the clinical setting only in the context of chemotherapy (14, 19, 20), we explored the effect in the presence of oxaliplatinum or irinotecan, two components of standard regimens for patients with colorectal cancer, at IC50 dosing established previously (Supplementary Figure 5). As expected, the stimulatory antibody effects were preserved in the presence of chemotherapy (Figure 2C, Supplementary Figure 6).

**Figure 3:**
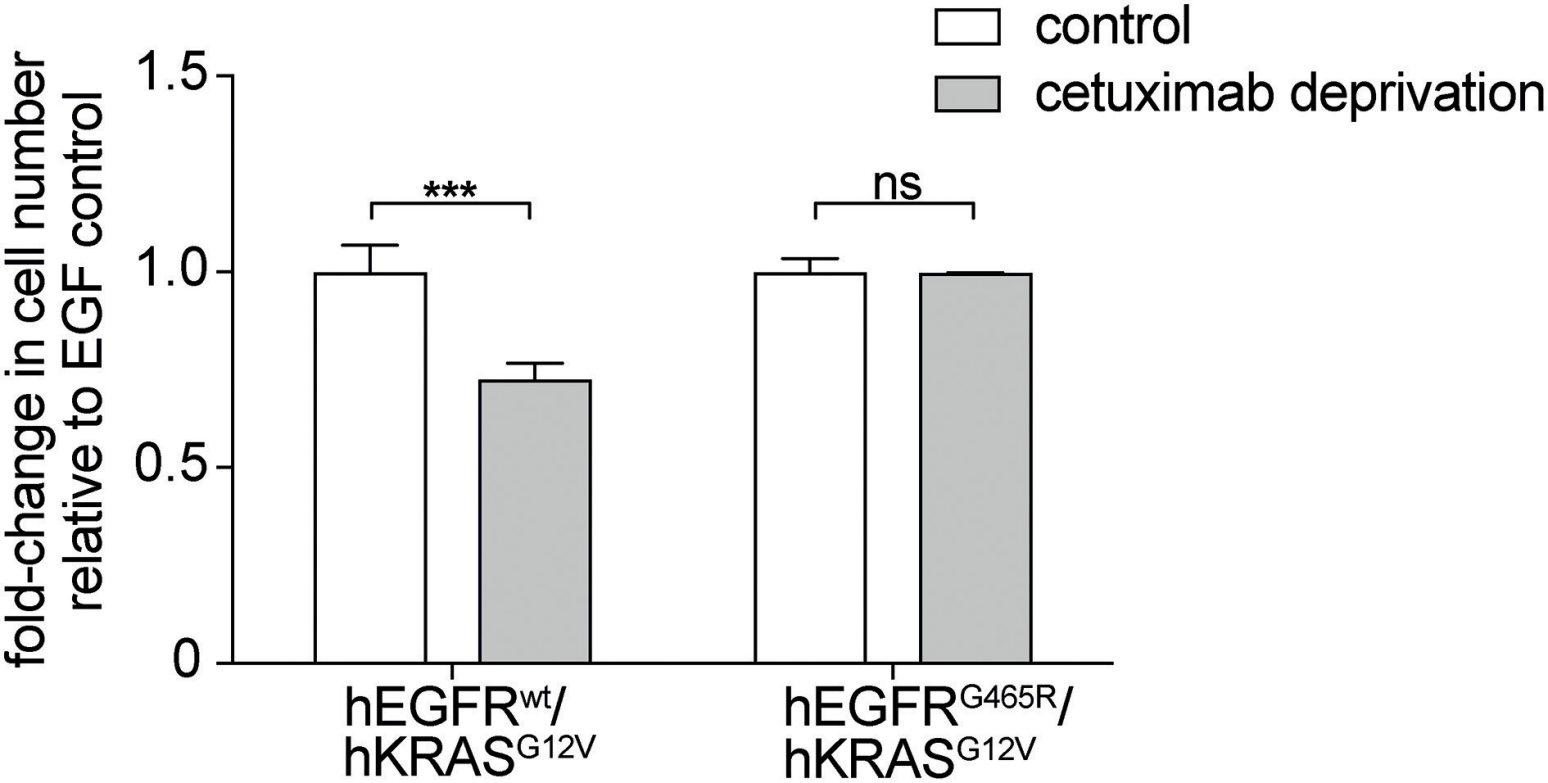
EGFR antibodies lead to drug-addiction phenotype of RAS mutant cells. hEGFR wt / hKRAS G12V Ba/F3 cells show restricted growth after antibody withdrawal. hEGFR wt / hKRAS G12V and hEGFR G465R / hKRAS G12V transduced Ba/F3 cells were treated with hEGF and cetuximab as indicated. Cells were seeded in triplicates at equal densities and the average number of viable cells was measured by trypan blue staining every 24 h for 7 days using Vi-CELL Cell Viability Analyzer. After 7 days, the therapeutic antibody was withdrawn and cells were washed three times with phospho-buffered saline (PBS). Cells were set back to the initial cell count of one million cells and incubated in hEGF-containing media for another week yet without cetuximab. For each treatment, data is expressed as the fraction of maximal cell count at 168 h normalized the hEGF-stimulated control. Results are represented as mean ± SD with (*n* = 3). Statistical significance was calculated using 2-way ANOVA followed by a Sidak *post-hoc* test for multiple comparison (***p < 0.001; ns, not significant).

Moreover, withdrawal of the EGFR antibody led to loss of the stimulatory effect on RAS mutant cells but only in an hEGFRwt context while EGFR mutated cells were not affected. This indicates that the stimulatory antibody effect is only transient (Figure 3). Interestingly, upon antibody withdrawal the proliferative capacity of the RAS mutant cells decreased even below the level of untreated cells indicating that these cells have become drug- (i.e., antibody-) addicted.

### Paradoxical Antibody Stimulation of RAS Mutant Cells Is Mediated by the EGFR Axis

**Figure 4:**
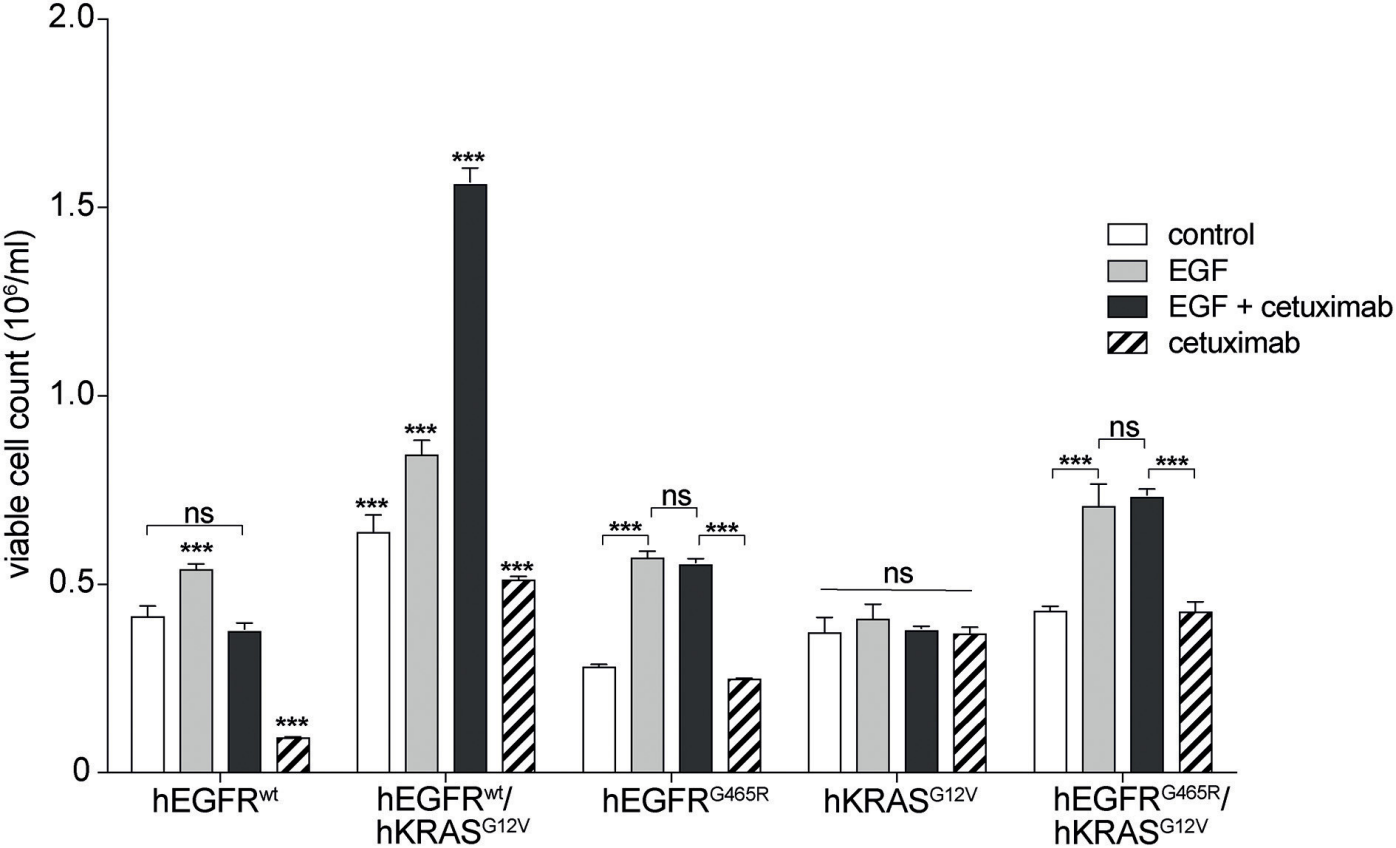
Paradoxical growth stimulation of RAS mutant cells by cetuximab requires EGFR binding. Proliferation of hEGFR wt, hEGFR wt / hKRAS G12V, hEGFR G465R, hKRAS G12V, and hEGFR G465R / hKRAS G12V transduced Ba/F3 cells was assessed in the absence of hEGF (control) or in the presence of hEGF, hEGF and cetuximab or cetuximab alone. Cells were seeded in triplicate at equal densities and cell viability was assessed by WST-8 assay. For each treatment, data are expressed as viable cell count at 168 h after initiation. Data is represented as mean ± SD with (*n* = 3). Statistical significance was calculated using 2-way ANOVA followed by a Tukey *post-hoc* test for multiple comparison (***p < 0.001).

Next, we wished to investigate if the paradoxical antibody-induced proliferation of RAS mutant cells resulted from antibody-EGFR interactions or from interactions of the antibody with potential other receptors on RAS mutant cells. We performed global kinase profiling using a PamStation®12 (PamGene Int.) to determine the differential kinase activity between Ba/F3 hEGFR wt / hKRAS G12V cells treated with hEGF or hEGF and cetuximab. These experiments showed differential kinase activity that was virtually limited to the MAPK pathway and we found no evidence for potential other pathways that would point to cetuximab off-target binding and pathway activation (data not shown). To more directly proof this, we studied the effect of cetuximab on hKRAS G12V expressing cells that either lack upstream EGFR or that express an hEGFR variant incapable of binding cetuximab (hEGFR G465R). Indeed, the stimulatory effect could only be produced in cell lines expressing hEGFR capable of binding cetuximab (Figure 4). This once more confirmed that the stimulatory effect of the EGFR antibody was not an off-target effect mediated by another cell surface receptor.

### Antibody Treatment of RAS Mutant Cells Paradoxically Sustains MAPK Signaling Downstream of RAS

**Figure 5:**
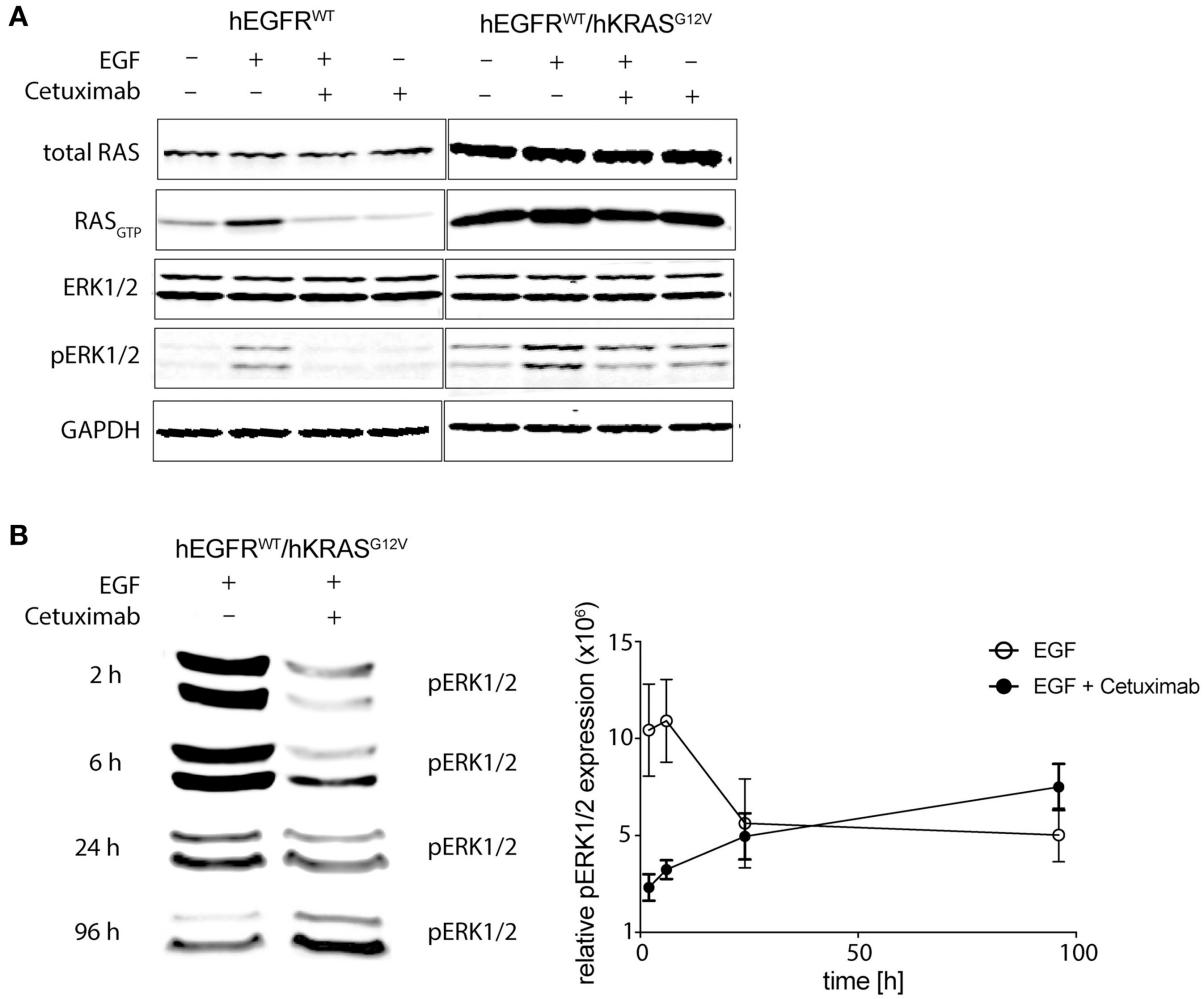
Cetuximab treatment sustains MAPK signaling in RAS mutant cells. **(A)** Short-term effects of cetuximab treatment on RAS_GTP_ loading and ERK phosphorylation in RAS mutated and non-mutated cells. hEGFR wt or hEGFR wt / hKRAS G12V transduced Ba/F3 cells were cultured with or without hEGF (5 ng/ml) and/or cetuximab (5 μg/ml) for 2 h. Protein from whole cell lysates was either directly subjected to Western Blot for total RAS, ERK1/2, phospho-ERK1/2, and GAPDH as a loading control or RAS_GTP_ was precipitated by GTP pulldown followed by SDS-PAGE before Western Blot was performed. The Odyssey CLx Infrared Imaging System was used for signal detection and quantification. One representative experiment out of three is shown. **(B)** Long-term effects of EGF and cetuximab treatment on ERK phosphorylation in RAS mutated cells. Experiments were performed as in **(A)** described, except cells were treated for 2, 6, 24, or 96 h before protein was obtained. Further, only ERK1/2 phosphorylation (pERK1/2) of hEGFR wt / hKRAS G12V transduced Ba/F3 cells treated with hEGF (5 ng/ml) alone or together with cetuximab (5 μg/ml) is shown. Western Blot of one representative experiment is shown and pooled data from three to four experiments was quantified using Fiji version 2.0.0-rc-46/1.5 g (ImageJ, Maryland, USA) and shown in the graph. Results are represented as mean ± SEM with (*n* = 3–4).

We could already describe the interference of EGFR phosphorylation by cetuximab and panitumumab in hEGFR wt in contrast to hEGFR G465R mutant Ba/F3 cells when EGF is present (28). We next wanted to investigate the effect of cetuximab/hEGF treatment on signal transduction via the RAS/MAPK axis in Ba/F3 cells expressing hEGFR in comparison to hEGFR / hKRAS G12V. First, Ba/F3 cells transduced with hEGFR wt and hEGFR wt / hKRAS G12V were checked for expression of murine RAS isoforms by targeted next-generation sequencing of both genomic and cDNA revealing wild-type status of all three murine isoforms (*KRAS, NRAS, HRAS*) (Supplementary Table 1). Next, we assessed the activation status of all RAS proteins by RAS-GTP pulldown assays as well as MAPK signaling downstream RAS (pERK) in both cell lines under different treatment conditions. In the basal state, hEGFR wt expressing Ba/F3 cells did not show substantial RAS_GTP_ loading nor ERK phosphorylation. As expected, hEGF stimulation of these cells led to ligand-mediated MAPK pathway activation as evidenced by an increase in ERK phosphorylation compared to untreated control, whereas concurrent treatment with cetuximab blocked hEGF-induced activation of ERK as well as RAS_GTP_ loading (Figure 5A). hEGFR wt / hKRAS G12V expressing Ba/F3 cells showed basal ERK phosphorylation even in the absence of growth factor, owing to the constitutive activation of RAS. Enhanced ERK phosphorylation levels and RAS_GTP_ loading was seen in response to hEGF stimulation indicating ligand-mediated activation of the remaining wild-type RAS isoforms in line with previously published data (25). Importantly, RAS mutant cells treated with hEGF and cetuximab showed sustained pERK levels that—in the course—exceeded those achieved by ligand stimulation alone, reflecting the proliferative characteristics of these cells (Figure 5B). We hypothesized that changes in pERK levels after Cetuximab withdrawl (as shown in Figure 3) explained the drug addiction phenotype. Indeed, when the hEGFR wt / hKRAS G12V Ba/F3 cells were deprived of the antibody after 1 week of treatment, ERK phosphorylation decreased (Supplementary Figure 7).

**Figure 6:**
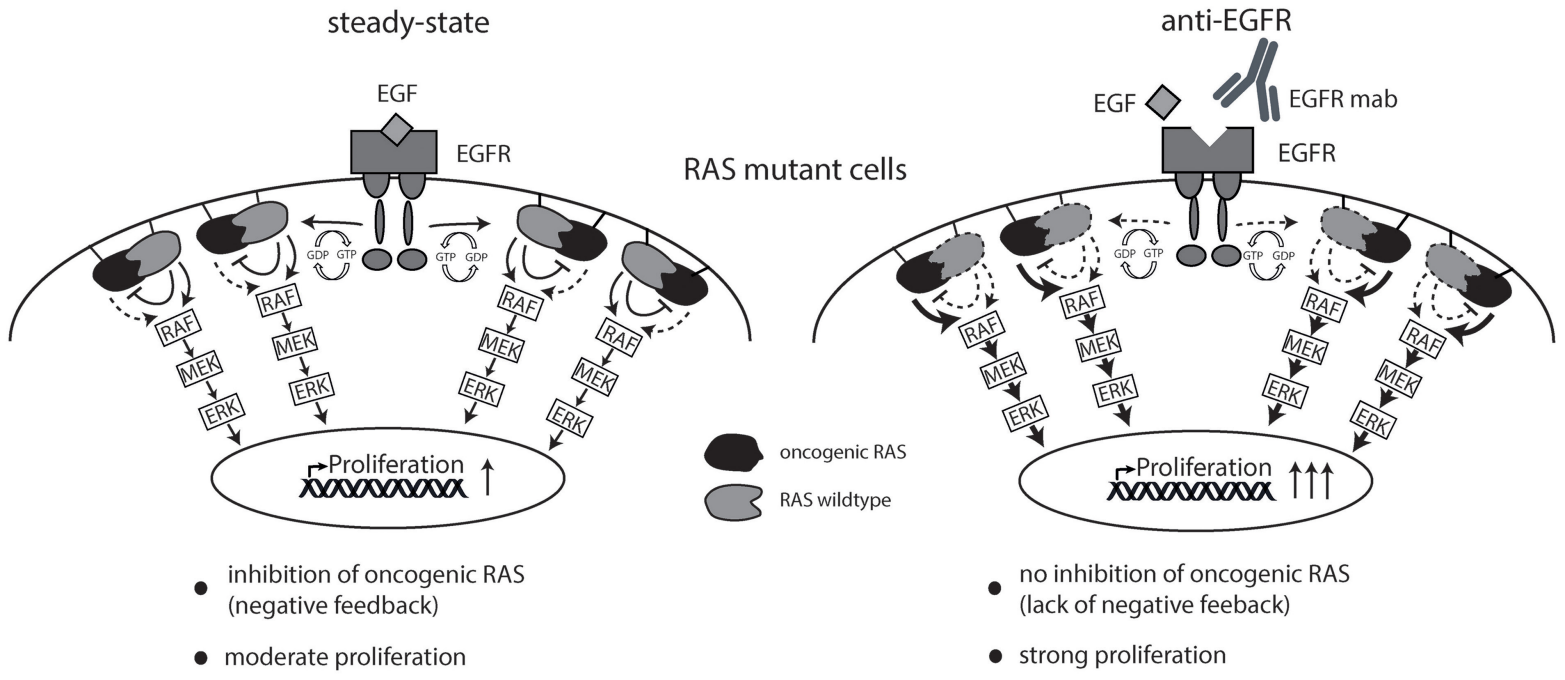
Schematic overview of working hypothesis. Strong ligand-mediated pathway activation of RAS mutated cells attenuates cellular growth and proliferation via RAS-isoform mediated negative feedback inhibition (left graphics). Cetuximab is able to limit ligand-receptor interaction and thereby prevents feedback inhibition on oncogenic RAS resulting in enhanced cellular growth and proliferation (right graphics).

Together, this data argues in favor of the hypothesis that EGFR inhibition sustains signaling downstream of oncogenic RAS by suppressing RAS inhibitory non-mutant isoforms thereby creating a drug-addiction phenotype (schematically illustrated in Figure 6).

## Discussion

RAS-mediated resistance represents a relevant clinical problem in the treatment of patients with colorectal cancer. Despite intensive research efforts, the complexity of clonal selection and the effects of EGFR-directed antibodies in the setting of oncogenic RAS mutations are still insufficiently understood. In patients with primary oncogenic RAS mutations, treatment with EGFR-inhibiting antibodies seems to worsen the prognosis compared to chemotherapy alone (14, 19, 20). At the same time, patients with RAS wt tumors at diagnosis tend to develop RAS mutant subclones that emerge under the selective pressure of EGFR antibodies, but seem to disappear after cessation of antibody treatment (22). Understanding the mechanisms underlying these clinical observations is necessary to develop alternative strategies to overcome acquired resistance to EGFR targeting in colorectal cancer.

Here, we used an EGF-dependent cell line to explore the molecular consequences of EGFR inhibition with monoclonal antibodies in the context of an oncogenic RAS mutation. Despite this simplistic model, we observed several effects that may explain the *prima vista* paradoxical clinical observations. In the presence of EGF that is also present in the tumor microenvironment, EGFR-directed antibodies showed a paradoxical stimulatory effect on RAS mutant cells. Such direct stimulatory effect of the antibody may not only underlie its detrimental effect in patients with RAS mutant tumors but may also account for the higher frequency of acquired RAS-mediated resistance as compared to resistance mediated by acquired EGFR ectodomain mutations. Our data clearly shows that tumor clones with EGFR ectodomain mutations are not sensitive to EGFR targeting, but don't seem to be stimulated by the antibody and therefore may have a lesser growth advantage than RAS mutant subclones during EGFR-directed therapeutic pressure. Moreover, we observed that after antibody withdrawal, the proliferative drive of RAS mutant cells falls below that of cells previously not exposed to the antibody. This suggests that RAS mutant cells become antibody-addicted resulting in lesser proliferation after antibody withdrawal. In accordance with this, previous studies have shown that acquired RAS mutant subclones decline after antibody withdrawal in colorectal cancer and there is further evidence for drug addiction mechanisms in melanoma on MAPK inhibitory treatment (22, 31). These findings open up perspectives of re-sensitizing RAS mutant tumors to EGFR inhibition by “drug holiday” concepts that remain to be clinically evaluated in prospective trials. Since EGFR ectodomain mutant subclones are not paradoxically stimulated by EGFR-directed antibodies, clonal frequencies of such acquired resistant clones should not decline after antibody withdrawal (as shown in our model system), very likely impeding re-sensitization in this resistance setting. Drug holidays to suppress RAS mutant tumor subclones could be targeted at “microscopic” resistance either using fixed duration or individual schedules tailored according to mutational RAS load (liquid biopsy) with the ultimate goal to achieve longer duration of response or disease control. Re-sensitization may, however, also be possible after overt clinical progression on EGFR-directed antibodies at second relapse as currently tested in clinical trials (e.g., NCT02934529).

Our model not only reflects some of the clinical observations of EGFR inhibition in the context of mutant RAS, it also offers insight into the mechanism underlying the paradoxical antibody stimulation. First, our data clearly shows that the monoclonal EGFR antibodies induce cell proliferation by interacting with the EGFR itself, since in functional receptor variants showing abrogated cetuximab and panitumumab binding the stimulatory effect is not seen. Therefore, the effect cannot be explained by off-target effects, e.g., by cetuximab binding and paradoxical stimulation of Insulin-like growth factor 1 receptor, a mechanism that has been proposed in the context of cetuximab resistance in gastric cancer (32). Our experiments dissecting RAS_GTP_ loading and downstream MAPK signaling are—in contrast—well-compatible with recent work suggesting that unmutated RAS isoforms mediate an inhibitory effect on oncogenic RAS (24–26, 33). This antagonism may be seen as a negative feedback loop that prevents overstimulation of the EGFR axis. While Young et al. (25) show that silencing of such inhibitory unmutated RAS isoforms releases signaling downstream oncogenic RAS, our data for the first time demonstrates that the inhibition of non-mutated RAS isoforms by monoclonal antibodies has the same effect. Interestingly, we observed no stimulatory effect, when the therapeutic antibody was used alone (in the presence of minimal, outcompeted concentrations of FBS-derived ligand), and—in the presence of high EGF concentrations—the size of the stimulatory effect increased with higher ligand to antibody ratios. This indicated that the stimulatory effect occurs only in the presence of low-level activation of RTK-mediated signaling, but not in the absence of such signals. In line with this, treatment with EGFR tyrosine kinase inhibitors did not produce stimulating effects in our model, since even low concentrations led to full blockade of the signaling axis. As a consequence, high-affinity EGFR antibodies should exert less stimulatory and therefore less harmful effects on RAS mutant tumor clones than lower-affinity therapeutic antibodies that permit residual RTK-mediated signaling. However, harmful effects have also been observed in some clinical trials with the high-affinity antibody panitumumab, suggesting that even in this setting residual RTK-mediated signaling may occur. This is on the other hand not surprising given the relatively low penetration of full-size monoclonal antibodies into the tumor core (34, 35) likely producing higher ligand to antibody ratios in large parts of the tumor. Moreover, panitumumab has a low potential to compensate any pathway stimulatory effects by other effector functions due to its limited antibody-dependent cytotoxicity as IgG2 antibody (36).

Intriguingly, paradoxical stimulatory effects are not restricted to the inhibition of wild-type RAS isoforms but seem to be a more common theme since they can also be produced by inhibition of unmutated isoforms of other signaling components of the RAS-RAF-MEK-ERK axis in the context of mutant RAS. Most prominently, it has been found that in tumors and normal cells with wild-type RAF, BRAF inhibitors stimulate ERK signaling in a RAS-dependent manner (37–41). This paradoxical activation is thought to explain why those drugs may induce cutaneous neoplasia or promote progression of RAS mutant leukemia (42).

Taken together, our data highlights the importance of understanding pathway signaling in clinical practice and of genotyping tumors prior to and while administering EGFR-inhibiting antibodies to identify patients who may experience adverse effects. Moreover, our data may shape our ideas about re-sensitizing RAS mutant tumors toward EGFR inhibition and opens up new perspectives for designing more resistance preventive treatment approaches such as drug holiday concepts for patients with metastatic colorectal cancer.

## Data Availability Statement

All datasets generated for this study are included in the article/Supplementary Material.

## Author Contributions

MB, AS, and CB designed the study. FB, BF, KR, MK, and SM established Ba/F3 model cells and viral vectors. SM, JT, MT, AO, LW, and CS performed the experiments. MB, JT, AO, MT, SM, and MK analyzed the data. The manuscript was drafted and written by JT, SM, MT, AO, and MB.

### Conflict of Interest

The authors declare that the research was conducted in the absence of any commercial or financial relationships that could be construed as a potential conflict of interest.
