# Supplementary material for: Cancer Cells Expressing Oncogenic Rat Sarcoma Show Drug-Addiction Toward Epidermal Growth Factor Receptor Antibodies Mediated by Sustained MAPK Signaling
Source: Front Oncol. 2020 Jan 21;9:1559. doi: 10.3389/fonc.2019.01559 (PMC6985072; doi:10.3389/fonc.2019.01559)
Supplement: Supplementary file 1 [file Table_1.docx]

Supplementary Material

# Supplementary Figures and Tables

| **Supplementary Table 1: Next-generation sequencing to determine expression and exon 2 mutation status of murine RAS isoforms.*** | | | | | | |
| --- | --- | --- | --- | --- | --- | --- |
|  | *KRAS* | | *NRAS* | | *HRAS* | |
|  | gDNA | cDNA | gDNA | cDNA | gDNA | cDNA |
| EGFR wt | wt | wt | wt | wt | wt | wt |
| EGFR wt / KRAS G12V | wt | wt | wt | wt | wt | wt |
| *hEGFR wt or hEGFR wt / hKRAS G12V transduced Ba/F3 cells were sequenced and analyzed for KRAS, NRAS and HRAS wt status as described in methods. gDNA: genomic DNA; cDNA: complementary DNA; wt: wild-type | | | | | | |

| **Supplementary Table 2: Primers for site-directed mutagenesis and In-Fusion cloning of hEGFR G465R mutant** | | |
| --- | --- | --- |
| **template** | **primer name** | **primer sequence 5'-3'** |
| hEGFR wt | **hEGFR_hG465R-mutagen_fw** | GTGATGGAGATGTGATAATTTCAAGAAACAAAAATTTGTGCTATGC |
| hEGFR wt | **hEGFR_hG465R-mutagen_rv** | GCATAGCACAAATTTTTGTTTCTTGAAATTATCACATCTCCATCAC |
| hEGFR start | **InFusion_hEGFR-fw** | GGATCCAAGGCCTGCGGCCGCATGCGACCCT |
| hEGFR end | **InFusion_hEGFR-rv** | CGTTAACACCGGTTCTAGATCATGCTCCAATAAATTCACTGCTTTG |
| wt: wild-type; fw: forward primer; rv: reverse primer; | | |

| **Supplementary Table 3: Next-generation sequencing primers for murine RAS isoforms** | | |
| --- | --- | --- |
| **template** | **primer name** | **primer sequence 5'-3'** |
| murine KRAS/NRAS | **Seq_*KRAS*_cgDNA_fw** | ACACTCTTTCCCTACACGACGCTCTTCCGATCTTATAAACTTGTGGTGGTTGGAGC |
| murine KRAS/NRAS | **Seq_*KRAS*_cgDNA_rv** | TGACTGGAGTTCAGACGTGTGCTCTTCCGATCTTAGCTGTATCGTCAAGGCAC |
| murine HRAS | **Seq_*HRAS*_cgDNA_fw** | ACACTCTTTCCCTACACGACGCTCTTCCGATCTTACAAGCTTGTGGTGGTGGGCGC |
| murine HRAS | **Seq_*HRAS*_cgDNA_rv** | TGACTGGAGTTCAGACGTGTGCTCTTCCGATCTCAGCTGGATGGTCAGGGCAC |
| green: Illumina-specific; black: gene-specific; fw: forward primer; rv: reverse primer; | | |


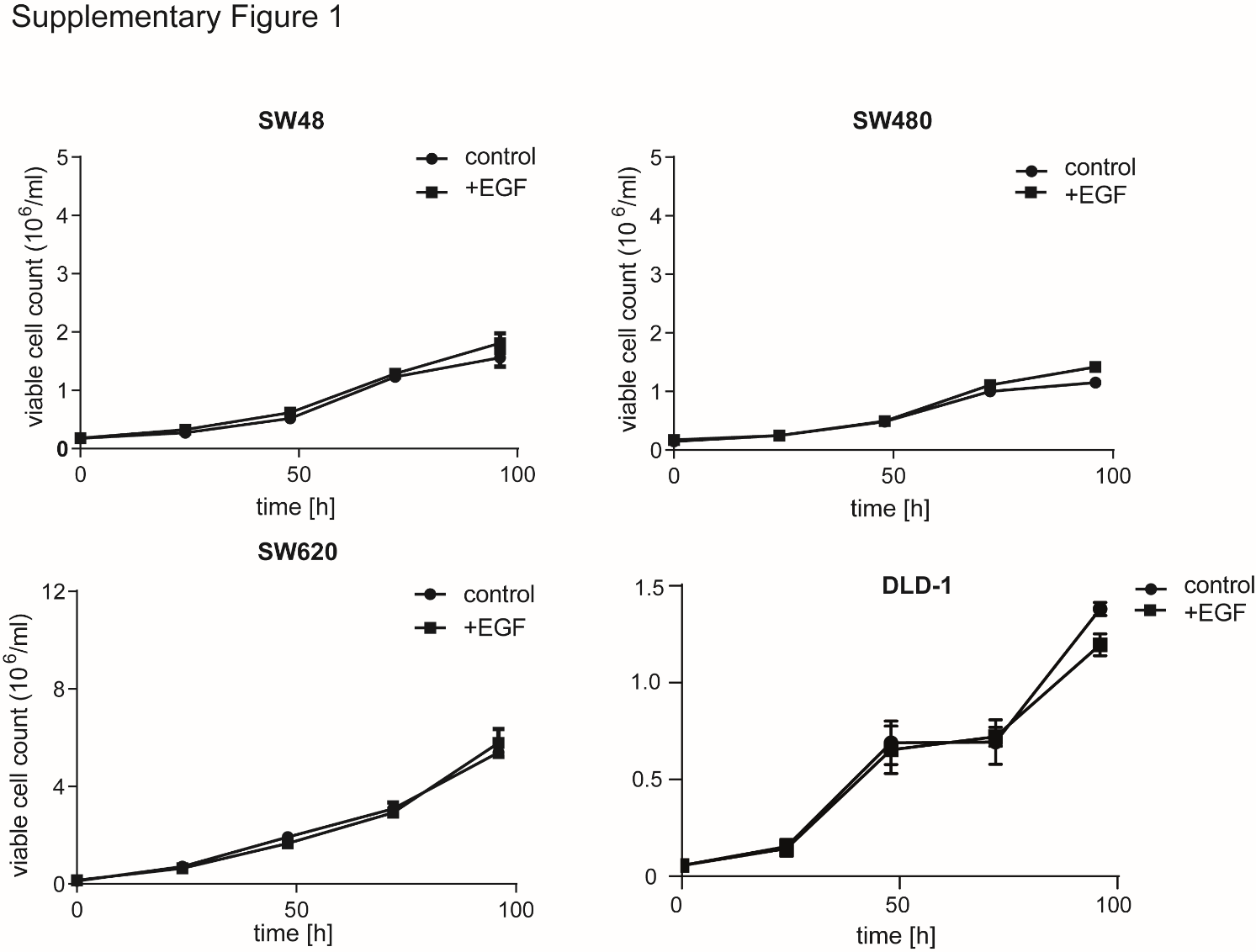


**Supplementary Figure 1: hEGF-independent growth of KRAS wild type and mutated cell lines.** Proliferation of wild-type KRAS SW48, KRAS G12V mutated SW480 and SW620 and KRAS G13V mutated DLD-1 cells was assessed in the absence and presence of hEGF. Cells were seeded in triplicate at equal densities and stimulated with hEGF 24 hours post-seeding. The average number of viable cells was measured by trypan blue staining for seven days using Vi-CELL Cell Viability Analyzer (Beckman Coulter, Brea, USA). Results of one representative experiment are shown as mean ± SD.

**
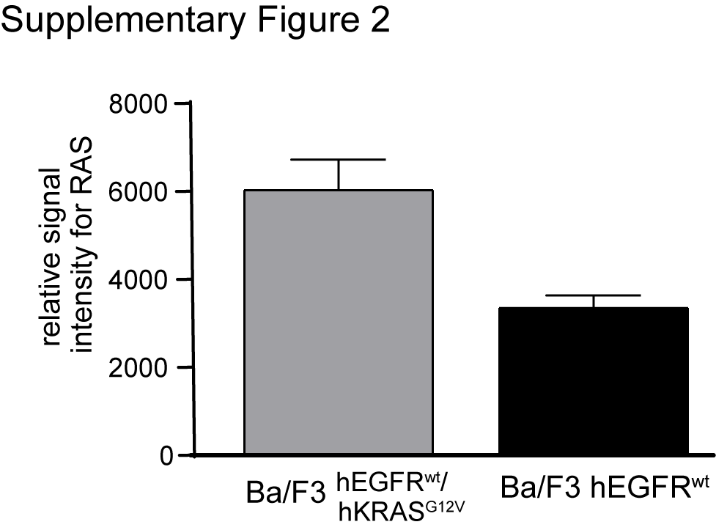
**

**Supplementary Figure 2: RAS expression in Ba/F3 cellular model.** Western Blots of Ba/F3 cell transduced with either hEGFR^wt^ or hEGFR^wt^/hKRAS^G12V^ grown with or without addition of hEGF/Cetuximab were quantified for total RAS levels. The figure shows RAS levels in cell lines used for further experiments.

**
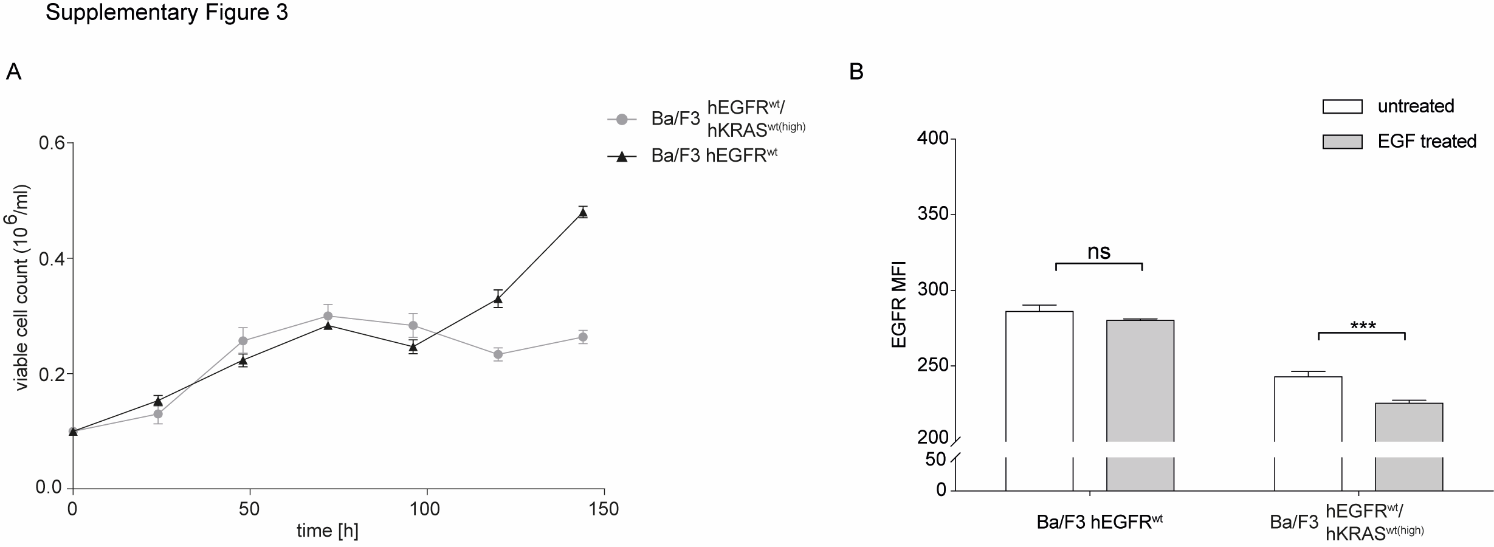
**

**Supplementary Figure 3: hEGFR wt / hKRAS wt (high) transduced Ba/F3 cells downregulate EGFR under EGF stimulation. (A)** hEGFR wt or hEGFR wt / hKRAS wt (high) transduced Ba/F3 cells were stimulated by EGF and cell count was measured every 24h. **(B)** Ba/F3 cells expressing hEGFR wt or hEGFR wt / hKRAS wt (high) were grown for 120h with or without addition of hEGF. Cells were then fluorescently labelled with EGFR antibody and specific secondary antibody and MFI was determined by FACS on a FACSCanto. Experiments were performed two times. Results are presented as mean ± SD. Statistical significance was calculated using Unpaired student’s t-test (B) (***: p<0.001; ns: not significant).


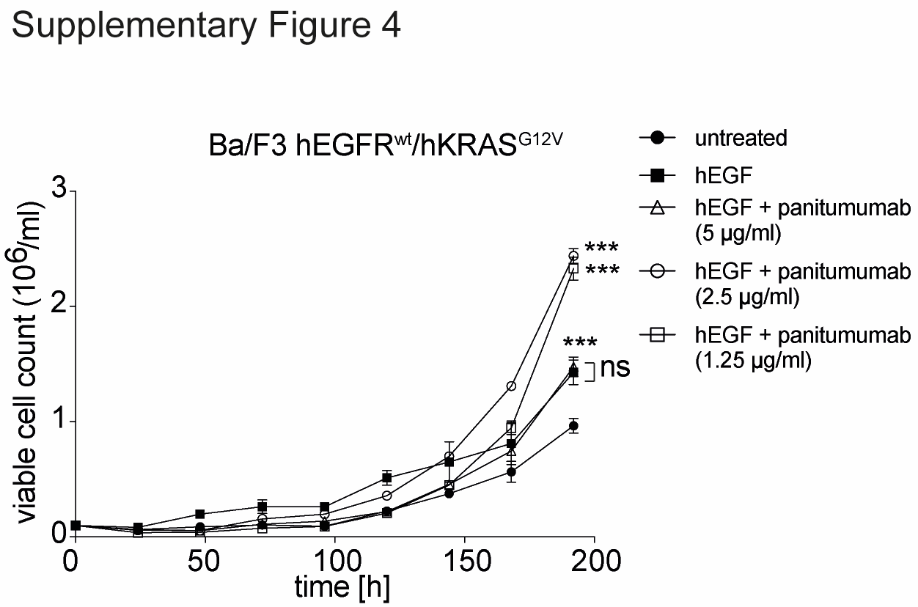


**Supplementary Figure 4: Paradoxical growth stimulation of RAS mutant cells by panitumumab.** Proliferation of hEGFR wt / hKRAS G12V transduced Ba/F3 cells was assessed in the absence or presence of hEGF plus 1.25, 2.5 or 5 µg/ml panitumumab as indicated. Cells were seeded in triplicates at equal densities and the average number of viable cells was measured by trypan blue staining every 24 hours for seven days using Vi-CELL Cell Viability Analyzer (Beckman Coulter, Brea, USA). For each treatment, data are expressed as the number of viable cells at the indicated time points. Results are shown as means ± SD. Statistical significance was calculated using a 2-way ANOVA test followed by a Tukey test for multiple comparison (***: p<0.001; *: p<0.05; ns: not significant).


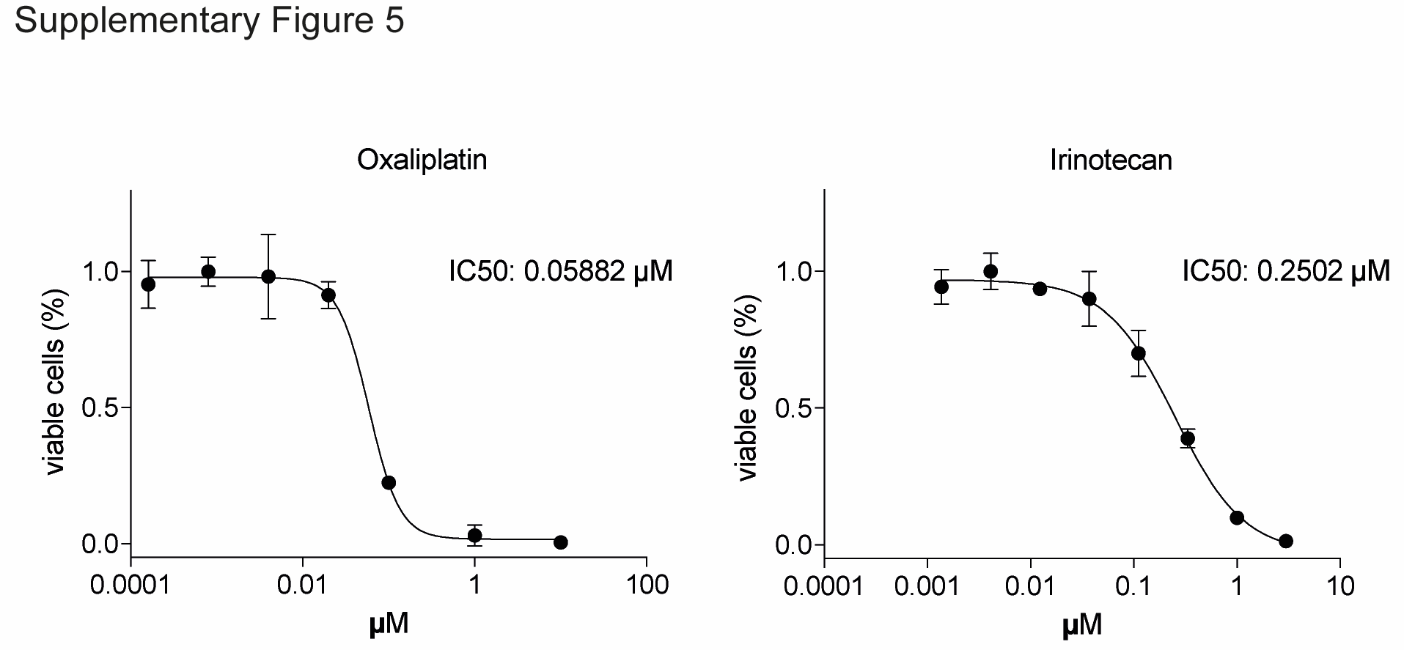


**Supplementary Figure 5: IC50-values of oxaliplatinum (left panel) and irinotecan (right panel) in hEGFR wt / hKRAS G12V transduced Ba/F3 cells.** Cells were seeded in triplicates at equal densities and treated with various amounts of cytostatics as indicated. The average number of viable cells was measured by WST-8 assay after 72 hours. IC50 values were calculated using GraphPad Prism6 software. Results are shown as means ± SD.


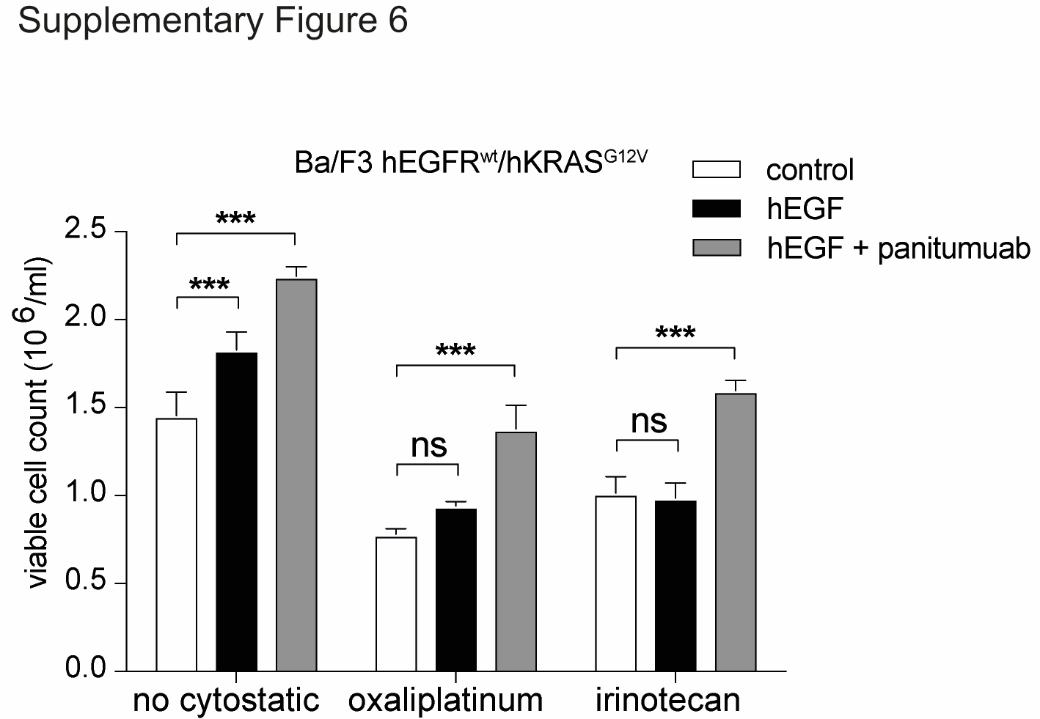


**Supplementary Figure 6: Stimulatory effect of panitumumab persists in the presence of oxaliplatinum and irinotecan.** hEGFR wt / hKRAS G12V transduced Ba/F3 cells were treated with hEGF, panitumumab and their representative IC50-value of oxaliplatinum and irinotecan as indicated. Cells were seeded in triplicates at equal densities and the average number of viable cells was measured by trypan blue staining every 24 hours for seven days using Vi-CELL Cell Viability Analyzer (Beckman Coulter, Brea, USA). For each treatment, data are expressed as viable cell count at 168 hours after initiation. The experiment was performed in triplicates and results are shown as means ± SD. Statistical significance was calculated using 2-way ANOVA followed by a Tukey test for multiple comparison (***: p<0.001; ns: not significant).

.


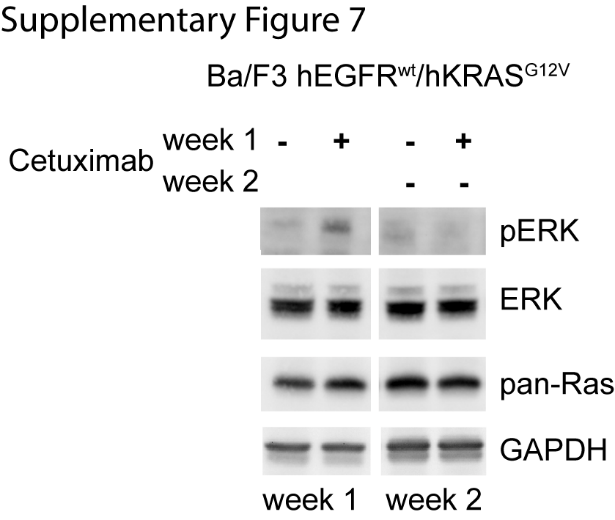


**Supplementary Figure 7: ERK phosphorylation decreases after Cetuximab withdrawl.** hEGFR wt / hKRAS G12V transduced Ba/F3 cells were treated with Cetuximab as described in Figure 3. Protein from whole cell extracts were subjected to Western Blot for pERK, ERK, pan-Ras and GAPDH.
