# Supplementary material for: Cancer Cells Expressing Oncogenic Rat Sarcoma Show Drug-Addiction Toward Epidermal Growth Factor Receptor Antibodies Mediated by Sustained MAPK Signaling
Source: Front Oncol. 2020 Jan 21;9:1559. doi: 10.3389/fonc.2019.01559 (PMC6985072; doi:10.3389/fonc.2019.01559)
Supplement: Supplementary file 2 [file Data_Sheet_1.docx]

## Supplementary Materials and Methods

*Cell lines.* Human colorectal cancer cell lines SW48 (ATCC CCL-231), SW480 (ATCC CCL-228) and SW620 (ATCC CCL-227) or DLD-1 (ATCC CCL-221) were cultivated in DMEM or RPMI respectively (both Life Technologies, Carlsbad, USA), supplemented with 10% (v/v) FBS (Merck Millipore, Billerica, USA) and 1% (v/v) penicillin/streptomycin (Gibco/Life Technologies) at 37°C in a humidified atmosphere including 5% CO_2_. Ba/F3 cells (CSC-C2045, Creative Bioarray, New York, USA) were kindly provided by Stefan Horn (UKE, Hamburg, Germany) and maintained in RPMI medium containing 10% (v/v) FBS, 1% (v/v) penicillin/streptomycin and 10 ng/ml recombinant murine interleukin-3 (IL-3) purchased from Peprotech (Rocky Hill, USA). *Mycoplasma* testing was done in June 2016 (Venor- GeM kit, Merck Millipore). For experiments, cells were used at passage 3 - 15 after thawing.

*Cloning of EGFR- and RAS expression constructs.* For ectopic expression of the complete human wild-type EGFR (hEGFR), the respective full-length cDNA was inserted into a third-generation, self-inactivating (SIN) lentiviral gene ontology vectors LeGO-iG3-Puro+/eGFP as well as LeGO-iG3-Puro+/mCherry as described previously (1,2). The hEGFR mutant G465R was generated from the wild-type construct in the pcDNA3.1(+) vector with the QuikChange XL Site-Directed Mutagenesis Kit (Agilent Technologies, Santa Clara, USA) as described (2), amplified using individually designed oligonucleotides (Supplementary Table 2) and cloned into the LeGO-iG3-Puro+/eGFP vector using the In-Fusion HD Cloning Plus (Takara Bio, Paris, France). The gene encoding wild-type human KRAS (hKRAS) was amplified from human colon Cancer cell line SW48 and hKRAS bearing an oncogenic mutation GGT>GTT that leads to the amino acid substitution G12V was amplified from human SW480 colorectal cancer cells and cloned into LeGO-iC2-Zeo+/mCherry. Vector maps and sequence data for all parental vectors are available at http://www.LentiGO-Vectors.de.

*Cell transduction with hEGFR and hKRAS expression constructs.* Ba/F3 cells were lentivirally transduced with wild-type or mutant hEGFR and/or wild-type hKRAS or hKRAS G12V encoding vector. Virus production was described earlier (1). Transduction efficiency was determined and transduced cells were sorted based on their selective fluorescent marker by fluorescence-activated cell sorting (FACS) on a FACS Aria Illu (Becton Dickinson, Heidelberg, Germany). Polyclonal cell lines stably expressing hEGFR wild-type, G465R mutant and/or hKRAS wild-type or G12V were established by puromycin (1 µg/ml; InvivoGen, San Diego, USA) and zeocin (100 µg/ml; InvivoGen, San Diego, USA) selection. Cells were transformed to IL-3 independence by subsequent culturing in the absence of IL-3 and in the presence of 5 ng/ml human EGF (hEGF) and were used for drug-sensitivity experiments.

*Cellular drug-sensitivity and proliferation assays.* Cetuximab (Merck, Darmstadt, Germany) and panitumumab (Amgen, Thousand Oaks, USA) were purchased from the Hospital Pharmacy. Ba/F3 cells transduced with hEGFR and/or hKRAS encoding vectors were seeded in triplicate at equal densities of 1.0 x 10^5^ cells/ml and cultured in the absence or presence of hEGF in combination with 5 µg/ml cetuximab or 2.5 µg/ml panitumumab. The average number of viable cells was measured by trypan blue staining every 24 hours for seven days using Vi-CELL Cell Viability Analyzer (Beckman Coulter, Brea, USA). Ligand and therapeutics were added freshly every 24 hours. EGFR expression of Ba/F3 cells was assessed by flow cytometry using EGFR antibody (R&D Systems, Minneapolis, USA), and specific secondary antibody (R&D Systems, Minneapolis, USA). Mean fluorescent intensity was measured by FACS on a FACSCanto (Becton Dickinson, Franklin Lakes, USA). Cell viability was assessed by WST-8 assay (cell counting kit-8, Sigma-Aldrich, St. Louis, USA). For this assay, 20000 cells were seeded in 100 µl hEGF-containing culture medium with the respective growth factor and/or inhibitor in 96-well culture plates and incubated for 72 hours. Viable cell numbers were determined by adding 10 µl of WST-8 reagent and absorbance was measured after two to three hours at a wavelength of 450 nm with a reference wavelength of 650 nm using a microplate reader. For combination treatment of Ba/F3 hEGFR wt / hKRAS G12V with cytostatics, the median inhibitory concentration (IC50 value) of oxaliplatinum and irinotecan was also determined by WST-8 assay. To do so, 5000 cells were seeded in hEGF-containing culture medium with serially diluted cytostatics in 96-well culture plates. Medium-only and 100 µM cisplatinum containing wells were added as controls. Cytotoxicity was assessed after 72 hours by using WST-8 reagents.

For cell proliferation assay of adherent colorectal cancer cell lines, SW48, SW480 SW620 or DLD-1 cells were seeded in triplicate at equal densities of 5 x 10^4^ cell/well in 12-well format. Cells were stimulated with 5 ng/ml hEGF 24 hours post-seeding. Proliferation was assessed after washing cells with phosphate buffered saline (PBS) following trypsinization (0.25% trypsin, 0.02% EDTA). Proliferation of cells was assayed as described above by acquiring data at 1, 2, 4, 5 and 7 days after initiation of treatment.

*PamGene serine/threonine kinase array analyses*. The differential kinase activity between Ba/F3 hEGFR wt / hKRAS G12V cells treated with hEGF versus hEGF + cetuximab was estimated using a PamGene serine/threonine Chip according to the manufacturer's instructions. Briefly, 1 x 10^6^ cells were lysed in M-PER Mammalian Extraction Buffer (Pierce) and subsequently treated as described previously (3). Image analysis, signal quantification for kinomic analysis and data analysis was performed using the BioNavigator software v. 6.2 (PamGene).

*Targeted next-generation sequencing (NGS) of RAS exons.* To assess expression of murine RAS isoforms in Ba/F3 cell lines, murine KRAS, NRAS and HRAS exon 2 sequences were amplified from genomic DNA and cDNA in two consecutive PCR reactions using the proof-reading polymerase Phusion HS II (Thermo Fisher Scientific Inc., Wilmington, USA) including overhangs for hybridization of amplicons to the Illumina flow cell for sequencing-primer annealing essentially as previously described (2,4). All primers are shown in Supplementary Table 3. Amplicons were purified after agarose gel electrophoresis using the NucleoSpin Gel and PCR Clean-up kit (Macherey-Nagel, Düren, Germany). All amplicons were multiplex-sequenced with a 500-cycle single indexed (8 nucleotides) paired-end run on a MiSeq (Illumina, San Diego, USA) machine. Overlapping paired reads were merged using the software FLASH (v1.2.6) (5). Non-overlapping reads were excluded from further analysis. Usearch (v6.0.307) (6) was employed to dereplicate and cluster the merged reads. Sequences observed less than 30 times were discarded and the remaining sequences were clustered according to their similarity with reference RAS sequences.

*MAPK signaling.* hEGFR wt or hEGFR wt / hKRAS G12V transduced Ba/F3 cells were cultured with or without hEGF (5 ng/ml) in combination with cetuximab (5 µg/ml) for 2, 6, 24 or 96 hours. Protein from whole cell lysates was subjected to Western blot analysis using primary antibodies recognizing ERK1/2 (#9107) and pERK1/2 (Thr202/Tyr204) (#9101, Cell Signaling Technology, Danvers, USA)*,* and GAPDH (#32233) or β-Actin (#47778, Santa Cruz Biotechnology, Inc. Texas, USA) as a loading control. IRDye 680RD and IRDye 800CW conjugated secondary antibodies were obtained from LI-COR Biosciences (Lincoln, USA). The Odyssey CLx Infrared Imaging System (LI-COR Biosciences) was used for signal detection and quantification.

*GTP-RAS pulldown.* hEGFR wt or hEGFR wt / hKRAS G12V transduced Ba/F3 cells were cultured with or without hEGF (5 ng/ml), in combination with cetuximab (5 µg/ml) for 2 hours. Active, RAS_GTP_ was precipitated using GST-Raf-1-RAS binding domain fusion proteins according to the manufacturer’s protocol (PR-950, JenaBioscience, Jena, Germany). Total protein and pulldown samples were separated by SDS-PAGE, followed by Western Blot. Ectopic human KRAS as well as murine RAS isoforms (KRAS, NRAS, HRAS) were detected with a pan-RAS mouse monoclonal antibody. Total lysates were immunoblotted with anti-GAPDH (#32233, Santa Cruz Biotechnology, Inc. Texas, USA) as a loading control. Signal was detected as described above.

*Data evaluation and statistical analysis.* Data was plotted using GraphPad Prism version 7.00 (GraphPad Software, La Jolla California USA). Unpaired student’s t-test or two-way ANOVA followed by appropriate test for multiple comparison were applied for statistical evaluation with p-values being calculated using two-sided tests. Findings were considered significant for p<0.05 (*), very significant for p<0.01 (**) and highly significant for p<0.001 (***). In all experiments, data shows mean ± SD or SEM of representative or combined experiments performed in triplicates as indicated.

1. Weber K, Bartsch U, Stocking C, Fehse B. A multicolor panel of novel lentiviral ‘gene ontology’ (LeGO) vectors for functional gene analysis. Mol Ther [Internet]. The American Society of Gene Therapy; 2008;16:698–706. Available from: http://linkinghub.elsevier.com/retrieve/pii/S1525001616314514

2. Braig F, März M, Schieferdecker A, Schulte A, Voigt M, Stein A, et al. Epidermal growth factor receptor mutation mediates cross-resistance to panitumumab and cetuximab in gastrointestinal cancer. Oncotarget [Internet]. 2015;6:12035–47. Available from: http://www.oncotarget.com/fulltext/3574

3. Haetscher N, Feuermann Y, Wingert S, Rehage M, Thalheimer FB, Weiser C, et al. STAT5-regulated microRNA-193b controls haematopoietic stem and progenitor cell expansion by modulating cytokine receptor signalling. Nat Commun. The Author(s); 2015;6:8928.

4. Li X, Xu L, Li H, Zhao L, Luo Y, Zhu Z, et al. Cetuximab-induced insulin-like growth factor receptor i activation mediates cetuximab resistance in gastric cancer cells. Mol Med Rep. 2015;11:4547–54.

5. Magoč T, Salzberg SL. FLASH: Fast length adjustment of short reads to improve genome assemblies. Bioinformatics. 2011;27:2957–63.

6. Edgar RC. Search and clustering orders of magnitude faster than BLAST. Bioinformatics. 2010;26:2460–1.
